# A LC-MS/MS method with electrospray ionization and atmospheric pressure chemical ionization source for analysis of pesticides in hemp

**DOI:** 10.1186/s42238-021-00106-9

**Published:** 2021-12-13

**Authors:** Avinash Dalmia, Erasmus Cudjoe, Jacob Jalali, Feng Qin

**Affiliations:** 1 Perkin Elmer, Inc., 710 Bridgeport Ave., Shelton, CT 06484 USA; 2 Perkin Elmer, Inc., Woodbridge, ON L4L 8H1 Canada

**Keywords:** Hemp, Liquid chromatography-mass spectroscopy, Pesticides, Electrospray ion source, Atmospheric pressure chemical ionization source

## Abstract

**Background:**

Pesticide testing for hemp has traditionally focused on techniques like QuEChERS with dSPE and SPE which demand time-consuming sample preparation, typically resulting in poor recovery rates for some pesticides, and requires the use of both LC-MS/MS and GC-MS/MS based instruments to cover the analysis for all regulated pesticides. In this study, we describe a streamlined approach for working with LC-MS/MS featuring a dual electrospray ionization (ESI) and atmospheric pressure chemical ionization (APCI) sources using solvent extraction for faster and easier sample preparation and with 80–120% recovery for the analysis of all of 66 pesticides (regulated by California state in cannabis) with low detection limits in hemp.

**Methods:**

A simple solvent extraction with acetonitrile was used to extract pesticides from hemp. A LC-MS/MS system with dual ESI and APCI source was used to determine sensitivity for the analysis of 66 pesticides in hemp matrix, 62 pesticides were analyzed using an 18-min LC-MS/MS method with an ESI source and the other 4 pesticides were measured using a 6-min LC-MS/MS method with an APCI source.

**Results:**

The limit of quantitation (LOQ) of all 66 pesticides in hemp was in the range of 0.0025–0.1 μg/g which was well below the California state action limits of these analytes in cannabis products. A simple, fast, and cost-effective solvent extraction method was used for sample preparation to get good recovery in the range of 80–120% with RSD less than 20%. The unique ionization mechanism of chlorinated pesticides such as pentachloronitrobenzene using the LC-MS/MS system with an APCI source was elucidated. The proficiency test report generated with the LC-MS/MS method showed acceptable results for all of 66 pesticides in hemp with all of th *z* scores less than 2 with no false positives and negatives. The stability data collected over 5 days showed RSD less than 20% for 66 pesticides in hemp, and this demonstrated the robustness of the LC-MS/MS system used in this work.

**Conclusions:**

A LC-MS/MS method with dual ESI and APCI sources was developed for the analysis of 66 pesticides in hemp. The recovery of all pesticides from a hemp matrix was in the acceptable range of 80–120% with RSD less than 20%.

## Introduction

With the passing of the 2018 Farm Bill legalizing hemp, farmers are allowed to grow hemp (https://www.brookings.edu/blog/fixgov/2018/12/14/the-farm-bill-hemp-and-cbd-explainer/ n.d.). This is good news for farmers—especially tobacco growers dealing with declining demand for their crop. Hemp, which can be used for fiber in textiles, is a member of the cannabis species but contains less than 0.3% tetrahydrocannabinol (THC), which gives users a high, and also high percentage of cannabidiol (CBD), which is purported to have multiple uses such as medical therapeutic benefits for patients with epilepsy, pain, nausea, and other medical disorders (Klein and Newton 2007). Like any other agricultural crop, pesticides are applied to hemp plants to protect it from pests and improve growth yield. Chronic exposure to pesticides can lead to serious health risks, and therefore, pesticide analysis in hemp is important for consumer safety and quality control.

Since there is no federal guidance for the analysis of pesticides in cannabis products such as cannabis flower, hemp, and others, different states in the USA have developed their own testing guidelines. Oregon was the first state in the USA to come up with comprehensive guidelines for pesticide residue analysis in cannabis products (Exhibit A 2017) and set regulatory limits for 59 pesticides in cannabis and cannabis-derived products. California, however, has issued more stringent action limits for 66 pesticides (including all but one of those found on Oregon state list and eight more), residues in cannabis flower, cannabis-infused edibles, and cannabis concentrates (Chapter 5 n.d.). According to Bureau of Cannabis text of regulations from California, 66 pesticides were divided into categories I and II. This document provided a guidance for action limit or limit of quantitation (LOQ) requirements for these 66 pesticides listed in two categories I and II in different cannabis-related products (Chapter 5 n.d.). According to the California state regulations, pesticides in category I should not be present in cannabis products, and these pesticides have LOQ limit of 0.1 μg/g, whereas pesticides in category II have specified different action limits or LOQ limits on inhalable and other cannabis products which are not inhaled. An action limit means that a recommended level of pesticide not to exceed in cannabis products.

Many past reports on pesticide analysis in cannabis and hemp products have centered on using time-consuming sample preparation methods (QuEChERS with dSPE and SPE) with lower recoveries for some of the pesticides and require use of both LC-MS/MS- and GC-MS/MS-based instruments for analysis of all the pesticides (Stenerson and Oden 2018; Kowlaski et al. 2017; Wang et al. 2016; Moulins et al. 2018; Alder et al. 2006; United States Department of Agriculture Food Safety and Inspection Service, Office of Public Health Science 2018; Anastassiades et al. 2003). The complex sample preparation methods and use of both GC-MS/MS and LC-MS/MS for pesticide analysis increase cost and complexity and reduce sample throughput. Previously, we published a method for the analysis of pesticides regulated by the California state in cannabis flower using the LC-MS/MS method with dual electrospray ionization (ESI) and atmospheric pressure chemical ionization (APCI) source (Dalmia et al. 2018). In this work, we extended our published method to the analysis of all 66 pesticides (including very hydrophobic and chlorinated pesticides typically analyzed by GC-MS/MS) spiked in hemp samples, well below the action limits specified by the California state in cannabis products. A LC-MS/MS instrument with ESI and APCI sources and a simple solvent extraction method with excellent recoveries for 66 analytes in acceptable range of 80–120% was used for the analysis. Also, the performance of the LC-MS/MS method was checked by conducting a blind Emerald Scientific’s proficiency test for 66 pesticides in hemp. Although we limited this LC-MS/MS method applicability to 66 pesticides regulated by the California state in cannabis products in this study, this LC-MS/MS method with dual ESI and APCI sources and solvent extraction can be extended to the analysis of pesticides regulated by different US states and other countries such as Canada, Israel, and others in cannabis products.

## Experimental

### Apparatus

- LC System—QSight^®^ LX-50 LC (PerkinElmer, Shelton, CT).
- MS system——QSight™ 420 MS/MS detector with ESI and APCI source with HSID interface and Simplicity 3Q™ software platform (PerkinElmer, Shelton, CT).
- LC Column—Quasar SP Pesticides C18, 100 mm long, 4.6 mm ID, 2.7 μm particle size, and particle is superficially porous (PerkinElmer, Shelton, CT).
- Mixer—analog vortex mixer (VWR, Radnor, PA).
- Centrifuge—Eppendorf centrifuge 5430 (Eppendorf Co. Ltd).
- Polypropylene centrifuge tubes—15 mL and 50 mL (PerkinElmer, Shelton CT).
- Glass volumetric flasks—50 mL (VWR, Radnor, PA).
- Syringes—3-mL plastic syringes with Luer lock (Becton Dickinson, Fingerlakes, NJ).
- Filter—Nylon syringe filter, diameter 30 mm, 0.22 μm pore size (PerkinElmer, Shelton, CT).
- LC vials—2-mL amber glass (PerkinElmer, Shelton, CT).

### Reagents

- Water—Optima LCMS grade (Fisher Scientific, Pittsburg, PA)
- Solvents—acetonitrile and methanol, Optima LCMS grade (Fisher Scientific, Pittsburg, PA)
- Formic acid (99.5%)—Optima LCMS grade (Fisher Scientific, Pittsburg, PA)
- Ammonium formate (99%)—Optima LCMS grade (Fisher Scientific, Pittsburg, PA)
- Three pesticide standard mixtures containing 65 pesticides and two individual standards of two isomers of chlordane at a concentration level of 100 μg/mL in acetonitrile (Accustandard, New Haven, CT)

### Sample preparation method

For method development, ground hemp samples were obtained from Emerald Scientific. Below is the step by step sample preparation procedure with 10-fold dilution:

- For each sample, approximately 5 g of ground hemp was used as a representative of each sample batch. In our method, hemp was already received after grinding, and therefore, there was no need for further grinding. Note that hemp plant material would need to be ground to a smaller particle size less than 1 mm for the efficient extraction of pesticides if it was present in its native form.
- Weigh accurately 1 g of this ground hemp sample and place it into a 50-mL centrifuge tube.
- Spike 100 μL of internal standard solution. Twenty deuterated analogs of 20 out of 66 pesticides in the California state list were selected as internal standards to compensate mainly for ion suppression effects to improve the quantitative analysis as well as overall recovery and to correct for any minute analyte loss during sample preparation. The 20 internal standards were as follows: Atrazine-d5 (CDN isotopes), Acequinocyl-d25 (TRC Canada), Boscalid-d4 (TRC Canada), Captan-d6 (CDN isotopes), Carbaryl-d7 (CDN isotopes), Daminozide-d4 (CDN isotopes), Diazinon-d10 (CDN isotopes), Dichlorvos-d6 (CDN isotopes), Dimethoate-d6 (CDN isotopes), Fipronil-13C2,15N2 (TRC Canada), Malathion-d6 (CDN isotopes), Methylparathion-d6 (CDN isotopes), Myclobutanil-d9 (CDN isotopes), Pentachloronitrobenzene-13C6 (CIL), Permethrin-d5 (TRC Canada), Phosmet-d6 (TRC Canada), Piperonylbutoxide-d9 (TRC Canada), Pyridaben-d13 (CDN isotopes), Thiamethoxam-d3 (CDN isotopes), and Trifloxystrobin-d6 (TRC Canada). Dissolve the 100 mg of captan-d6 and 10 mg of other 19 internal standards into 1 mL of acetonitrile in different 10 mL tubes. Pipette 40 μL each of internal standard solutions into a 10-mL volumetric flask, and fill it to the mark with acetonitrile to give an internal standard solution containing captan-d6 at a concentration of 400 μg/mL and remaining 19 internal standards at a concentration of 40 μg/mL.
- Add 3 steel balls (10 mm in diameter) to the tube for efficient extraction during vortex mixing.
- Add 5 mL of LC/MS grade acetonitrile with 0.1% formic acid to the tube and cap it. Formic acid was added to acetonitrile to minimize the degradation of captan (Mastovska et al. 2004).
- Place the tube on a multi-tube vortex mixer and allow it to vortex for 10 min.
- Centrifuge extract in tube for 10 min at 3000 rpm.
- Filter the solvent into a 5-mL glass amber vial using a 0.22-μm nylon syringe filter and cap it.
- Label the bottle with the sample ID.
- Transfer 0.5 mL of extracted sample into a 2-mL HPLC vial and dilute it with 0.5 mL of LC/MS grade acetonitrile with 0.1% formic acid and mix it.

### Preparation of standard solutions and calibration standards

The stock pesticide standards come in a 100 μg/mL concentration in acetonitrile and to prepare 1 μg/mL intermediate standard, pipette 100 μL of stock standard from each pesticide ampule and transfer it to a volumetric flask and fill it to 10 mL level with acetonitrile containing 0.1% formic acid. This intermediate standard solution was stored at − 20 °C and was diluted in acetonitrile with 0.1% formic acid to get solutions over a concentration range of 1 to 100 ng/mL for the preparation of solutions for calibration curves and other studies such as recovery, ion suppression, and long-term stability. To produce a quantitative matrix-matched calibration curve, we spiked 980 μL of hemp extract with 10 μL of 100–10,000 ng/mL standards for pesticides to get the concentration of pesticides in the range of 1–100 ng/mL in hemp extract which is equal to 10–1000 ng/g in a hemp sample based on the overall dilution factor of 10 and 10 μL of internal standard solution.

### LC conditions

- Mobile phase for LC-MS/MS method with an ESI source. Solvent A (0.1% formic acid, 2 mM ammonium formate in water) was prepared by adding 1 mL of formic acid and 0.126 g of ammonium formate to 1 L of water. Solvent B (0.1% formic acid, 2 mM ammonium formate in methanol) was prepared by adding 1 mL of formic acid and 0.126 g of ammonium formate to 1 L of methanol. The LC gradient was 5% B for 0.5 min, increased linearly to 60% B in 3.5 min followed by a linear increase to 95% B in 8 min and from 95 to 100% B in 0.5 min and maintained at 100% B for 4 min. The column was re-equilibrated for 1.5 min with starting mobile phase before each injection. The run time for the optimized gradient elution method including analytical column re-conditioning was 18 min for the ESI method.
- Mobile phase for the LC-MS/MS method with APCI source. Solvent A is water, and solvent B is methanol. The LC gradient was 80% B for 0.5 min, increased linearly to 100% B in 1.5 min, and maintained at 100% B for 2.5 min. The column was re-equilibrated for 1.5 min with starting mobile phase before each injection. The run time for the optimized gradient elution method including analytical column re-conditioning was 6 min for the APCI method.
- Flow rate, 0.8 mL/min.
- Column temperature, 30 °C.
- Injection volume, 3 μL for the ESI method and 10 μL for the APCI method.
- Autosampler temperature, 2 mL LC Amber vials were maintained at 10 °C in the autosampler to prevent degradation of analytes.

### MS source conditions for ESI and APCI source

- ESI voltage, 5100 V in positive ion mode
- ESI voltage, − 4200 V in negative ion mode
- APCI corona discharge current, − 3 μA in negative ion mode
- ESI source temperature, 315 °C.
- APCI source temperature, 250 °C.
- Hot source induced desolvation (HSID) temperature for the ESI method, 200 °C
- HSID temperature for the APCI method, 180 °C
- Drying gas setting, 150 arbitrary units
- Nebulizer gas setting, 350 arbitrary units
- Acquisition mode—time managed multiple reaction monitoring (MRM) mode

A proficiency test is an interlaboratory test that allows the evaluation of the performance of their methods (Makkar et al. 2016). To demonstrate the accuracy and validation of our pesticide method, we participated in Emerald Scientific’s blind proficiency test for pesticides regulated by the state of California in hemp. About 50 different laboratories participated in this proficiency test for pesticides analysis in the hemp matrix. Each laboratory was provided with 1 g of hemp sample spiked with different amounts of pesticides and 1 g of blank hemp material and was asked to report the concentration of pesticide residues found in the provided sample with their methods. After our submission of proficiency test results for the hemp sample, Emerald Scientific calculated the average and standard deviation of results obtained from all of the laboratories. Conventional statistical methods were used to identify outliers from submitted data, and they were eliminated from this calculation. According to the international harmonized protocol of proficiency testing of analytical chemistry laboratories, *z*-score was used as a quantitative criterion for the evaluation of the performance of laboratory methods. The *z*-score for each pesticide in the spiked sample was calculated by dividing the absolute difference between a lab result and mean of all results by standard deviation of all the laboratories’ results. The following internationally accepted classification was used: *z* ≤ 2, satisfactory result; 2 < *z* < 3, doubtful result; and *z* > 3, unsatisfactory result (Makkar et al. 2016; ISO-13528 2005; Thompson et al. 2006).

## Results

### Detectability and reproducibility

Using a LC-MS/MS method, we were able to meet the California state regulatory action limits for all of 66 pesticides in hemp. The LOQ for all 66 pesticides was less than the California regulatory limits by a factor of 2–2000 in hemp. Using this method, 62 pesticides on the California list were analyzed using LC-MS/MS with an ESI source, and other 4 pesticides were measured using LC-MS/MS with an APCI source. The limits of quantification (LOQs) and response reproducibility at LOQ level for each of the pesticides (categories II and I) in hemp extract are summarized in Tables 1 and 2. The LOQs were determined by taking into account the signal of the quantifier ion (S/N > 10). The response RSD for each pesticide at its LOQ level in the hemp matrix was less than 20%. The retention time for each analyte was reproducible within ± 0.1 min over a 24-h period. This demonstrates that the method is more than adequately sensitive and reproducible for pesticides analysis in hemp at the regulatory limit specified by the state of California. The matrix-matched calibration curves showed excellent linearity for all analytes in the hemp matrix with a correlation coefficient (*R*^2^) greater than 0.99.

**Table 1:**
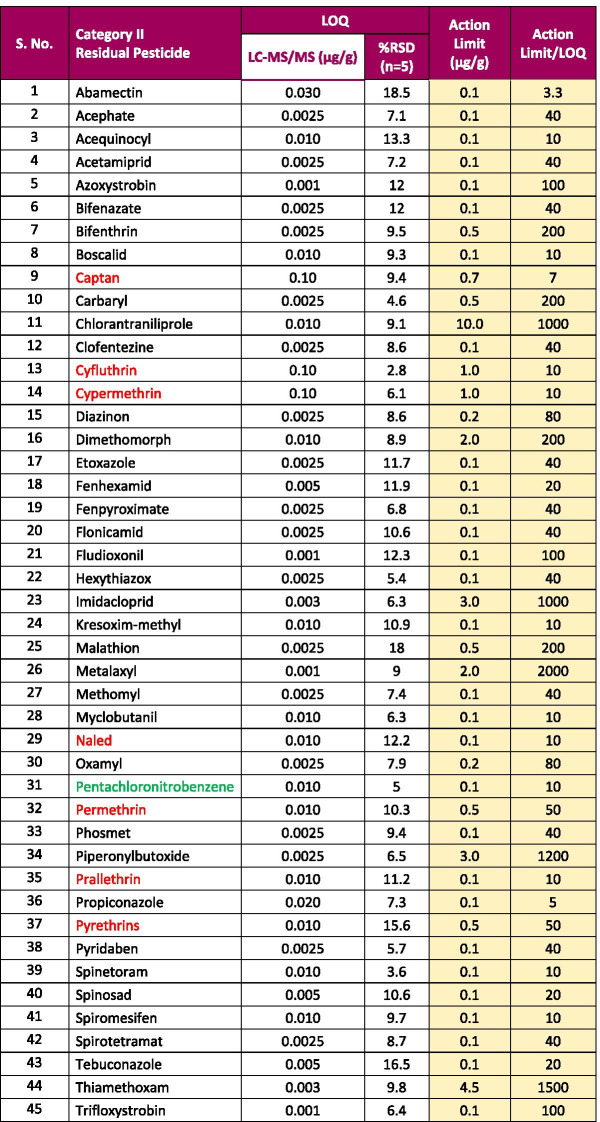
Limit of quantitation (LOQ), % RSD at the LOQ level, action limit, and ratio of action limit to LOQ for California category II pesticides with LC-MS/MS in hemp

Pesticides written in red/green: Pesticides typically analyzed by GC-MS/MS; pesticides written in black: pesticides typically analyzed using LC-MS/MS; pesticides written in red/black: pesticides analyzed using our LC-MS/MS method with electrospray ionization (ESI) source; and pesticides written in green: pesticides analyzed using our LC-MS/MS method with atmospheric chemical ionization (APCI) source

RSD stands for relative stand deviation of response for California category II pesticides at LOQ level in five (*n* = 5) hemp samples

**Table 2:**
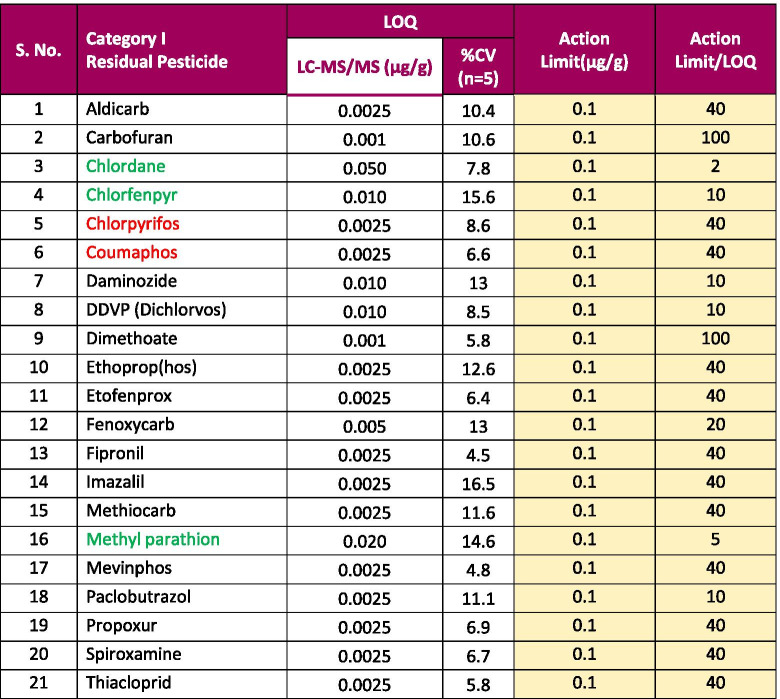
Limit of quantitation (LOQ), % RSD at the LOQ level, action limit, and ratio of action limit to LOQ for California category I pesticides with LC-MS/MS in hemp

Pesticides written in red/green: pesticides typically analyzed by GC-MS/MS; pesticides written in black: pesticides typically analyzed using LC-MS/MS; pesticides written in red/black: pesticides analyzed using our LC-MS/MS method with electrospray ionization (ESI) source; and pesticides written in green: pesticides analyzed using our LC-MS/MS method with atmospheric chemical ionization (APCI) source

RSD stands for relative stand deviation of response for California category II pesticides at the LOQ level in five (*n* = 5) hemp samples

### Recovery studies with solvent extraction

We used a simple acetonitrile-based solvent extraction method for the extraction of pesticides from the hemp matrix. To determine the recovery of pesticides with this method, fortified hemp samples were used to determine pesticides recovery. Three hemp samples were spiked at a low level of 0.1 μg/g for 66 pesticides. This level was chosen based on the lowest regulatory limits from California and other states in the USA for pesticides in cannabis- and hemp-related products. Tables 3 and 4 show that absolute recoveries of all 66 pesticides at a low level of 0.1 μg/g were within the acceptable range of 80–120% with RSD less than 20% for three hemp samples.

**Table 3:** Recovery of California category II pesticides at a level of 0.1 μg/g in hemp with solvent extraction

S. no.	Category II residual pesticide	Level 0.1 μg/g	
		Recovery/%	RSD/% (***n*** = 3)
1	Abamectin	100	16
2	Acephate	101	3
3	Acequinocyl	95	6
4	Acetamiprid	101	4
5	Azoxystrobin	104	4
6	Bifenazate	98	5
7	Bifenthrin	101	7
8	Boscalid	102	3
9	Captan	90	10
10	Carbaryl	99	4
11	Chlorantraniliprole	102	5
12	Clofentezine	94	6
13	Cyfluthrin	87	10
14	Cypermethrin	90	8
15	Diazinon	101	5
16	Dimethomorph	93	5
17	Etoxazole	98	5
18	Fenhexamid	92	5
19	Fenpyroximate	100	6
20	Flonicamid	100	3
21	Fludioxonil	102	5
22	Hexythiazox	96	6
23	Imidacloprid	97	4
24	Kresoxim-methyl	98	6
25	Malathion	99	5
26	Metalaxyl	103	5
27	Methomyl	99	3
28	Myclobutanil	100	5
29	Naled	100	5
30	Oxamyl	99	4
31	Pentachloronitrobenzene	90	6
32	Permethrin	97	7
33	Phosmet	102	5
34	Piperonylbutoxide	96	6
35	Prallethrin	88	7
36	Propiconazole	96	6
37	Pyrethrins	97	6
38	Pyridaben	99	6
39	Spinetoram	99	8
40	Spinosad	91	10
41	Spiromesifen	100	5
42	Spirotetramat	98	5
43	Tebuconazole	100	5
44	Thiamethoxam	97	3
45	Trifloxystrobin	99	6

RSD stands for relative standard deviation of recovery of California category II pesticides at a level of 0.1 μg/g for three (*n* = 3) hemp samples

**Table 4:** Recovery of California category I pesticides at a level of 0.1 μg/g in hemp with solvent extraction

S. no.	Category I residual pesticide	Level 0.1 μg/g	
		Recovery/%	RSD/% (***n*** = 3)
1	Aldicarb	99	3
2	Carbofuran	100	4
3	Chlordane	91	7
4	Chlorfenapyr	84	8
5	Chlorpyrifos	99	6
6	Coumaphos	99	5
7	Daminozide	74	3
8	DDVP (Dichlorvos)	95	5
9	Dimethoate	98	4
10	Ethoprop(hos)	99	5
11	Etofenprox	98	6
12	Fenoxycarb	100	6
13	Fipronil	102	6
14	Imazalil	95	6
15	Methiocarb	101	3
16	Methyl parathion	99	6
17	Mevinphos	100	4
18	Paclobutrazol	98	5
19	Propoxur	98	4
20	Spiroxamine	99	4
21	Thiacloprid	101	4

RSD stands for relative standard deviation of recovery of California category I pesticides at a level of 0.1 μg/g for three (*n* = 3) hemp samples

### LC-MS/MS method with optimum MRM transitions for challenging analytes in hemp

**Fig. 1:**
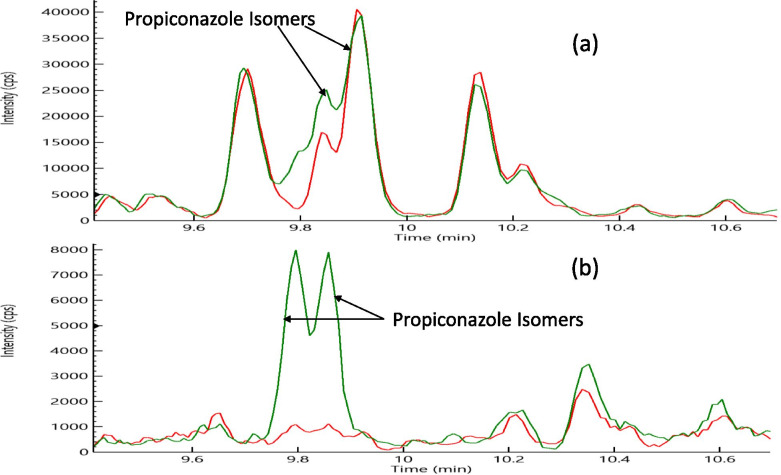
**a** Overlay of the response of blank hemp matrix (red) and propiconazole (green) at a level of 0.1 μg/g in the hemp matrix showing the matrix interference with a MRM transition of 342.1 to 69. **b** Overlay of the response of blank hemp matrix (red) and propiconazole (green) at a level of 0.1 μg/g in the hemp matrix demonstrating no matrix interference with a MRM transition of 344.1 to 69

The analysis of pesticide residues in hemp is a complex problem due to the concentration level disparities between naturally occurring cannabinoids and endogenous compounds such as terpenes in the range of 1–25% and incurred pesticide residues in the range of low ppb to ppm in the hemp matrix. Hemp is a difficult matrix to test for a low level of pesticides since it shows substantial matrix interference, caused by the presence of isobaric compounds, for the signal of some pesticides. To improve the selectivity of pesticides analysis in hemp, therefore, it is necessary to have multiple transitions for few compounds in order to find a transition that does not have matrix interference. For example, propiconazole can be ionized easily as a protonated molecular ion in a standard, but the MRM transition (342.1 to 69) in Fig. 1a, based on monoisotopic mass ion in the hemp matrix, showed poor LOQ of 0.5 μg/g due to matrix interference from coextracted compounds isobaric to this pesticide in hemp matrix. Therefore, as shown in Fig. 1b, MRM transition (344.1 to 69) based on M+2 isotope mass was determined to reduce matrix interference and achieve LOQ of 0.02 μg/g for propiconazole in the hemp matrix. Figure 1 shows the signal overlay of blank hemp matrix and propiconazole spiked at a level of 0.1 μg/g in hemp using MRM transitions with and without matrix interference. This figure demonstrates that optimum propiconazole MRM transition helped in achieving lower detection limits due to minimal matrix interference from hemp. Similarly, we had to determine the optimum MRM transitions for other pesticides such as acequinocyl, prallethrin, and pyrethrins to reduce matrix interference.

### Analysis of challenging analytes using the LC-MS/MS method with an ESI source

**Fig. 2:**
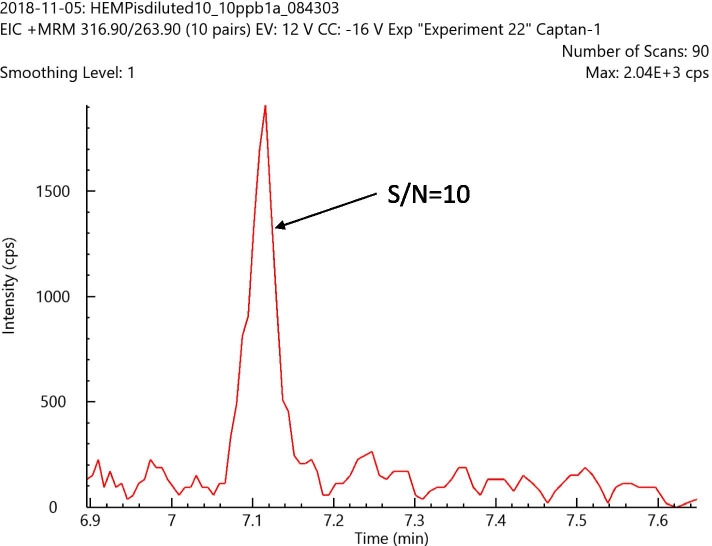
Response for captan with a signal to noise of 10 at a LOQ level of 0.1 μg/g in hemp using the LC-MS/MS method with an ESI source

A number of pesticides in cannabis and hemp, regulated by California and other states, are analyzed traditionally using GC-MS/MS with an EI source. Some examples of these pesticides analyzed normally with GC-MS/MS are cypermethrin, cyfluthrin, captan, naled, parallethrin, permethrin, pyrethrins, chlorpyrifos, and coumaphos. To achieve the required sensitivity, the selected MRMs and source conditions (source temperature and gas flow rate) were optimized with a heated electrospray source. LOQs for these analytes were in the range of 0.0025 to 0.1 μg/g, well below the California action limits in hemp. Among all analytes mentioned earlier, the analysis of captan using LC-MS/MS with an ESI source is most difficult. Figure 2 shows a good signal to noise of 10 for captan at LOQ level of 0.1 μg/g in hemp using the LC-MS/MS method with an ESI source and it can easily meet California state action limits of 0.7 μg/g for captan in hemp.

### Hydrophobic and non-polar pesticides analyzed with APCI

**Fig. 3:**
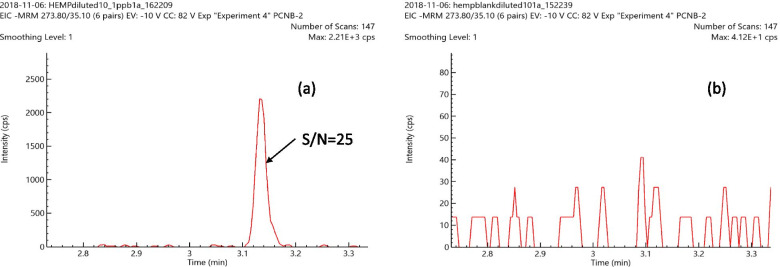
**a** The response for PCNB with a signal to noise of 25 in the hemp matrix spiked at a level of 0.010 μg/g. **b** PCNB response in the blank hemp matrix shows no matrix interference since matrix blanks show very little signal with random electrical noise and no matrix interference

Hydrophobic and non-polar pesticides (e.g., pentachloronitrobenzene, methyl parathion, chlordane, and chlorfenapyr) are traditionally analyzed by GC-MS/MS since they do not ionize effectively by LC-MS/MS with an ESI source. Since an APCI ion source is better suited for ionization of very hydrophobic and non-polar analytes, APCI was used to determine the detection limits of chlorfenapyr, pentachloronitrobenzene, methyl parathion, and chlordane in hemp. LOQ of pentachloronitrobenzene, methyl parathion, chlorfenapyr, and chlordane in hemp was in the range of 0.01–0.05 μg/g which is well below the California action limits for these pesticides in hemp. As a representative example for these four pesticides, Fig. 3a shows the excellent signal to noise (S/N = 25) for pentachloronitrobenzene (PCNB) spiked at a level of 0.010 μg/g in the hemp matrix using the LC-MS/MS system with an APCI source. This shows that the LC-MS/MS method with an APCI source for the analysis of PCNB in hemp is extremely sensitive and can easily meet the California state action limits. Based on FDA method validation guidelines to determine the selectivity of analysis, the acceptance criteria for selectivity is that matrix blanks should be free of any matrix interference peaks at the retention time of an analyte (Bioanalytical Method Validation Guidance for Industry 2018). In Fig. 3b, the blank hemp matrix response for PCNB shows a low background signal with random electrical noise and no matrix interference peak at the retention time of PCNB, and this demonstrates that the measurement of PCNB in the hemp matrix is quite selective. Similarly, the matrix blank signal for the other three pesticides (chlorfenapyr, methyl parathion, and chlordane) with an APCI source showed no matrix interference peaks at the retention time of these analytes, and the LC-MS/MS method with an APCI source showed a good signal to noise for the hemp matrix spiked with these pesticides at the level of California action limits of 0.1 μg/g or lower. The matrix-matched calibration curves for PCNB response in hemp showed excellent linearity over the concentration range of 1–1000 ng/g (corresponds to 10–10,000 ng/g in the hemp matrix) in 10× diluted hemp matrix extract with a correlation coefficient (*R*^2^) of 0.9991. Since the regression fit value for PCNB is greater than 0.99, it meets easily the requirement of the California bureau of cannabis control for regression fits to be higher than 0.99 (Chapter 5 n.d.). The accuracy of the calibration curve was checked by comparing back-calculated concentrations from the calibration curve with known concentrations of PCNB and the strict criterion of maximum deviation of 15% was met for all concentration levels.

### Ionization mechanism of PCNB with APCI source

For the ionization of compounds in the APCI source in negative ion mode, different ionization mechanisms such as proton abstraction, anion adduction, electron capture, and dissociative electron capture have been proposed in the past (McEwena and Larsen 2009). It has been demonstrated that chlorinated nitrobenzene compounds can form phenoxide ions under negative APCI conditions (Dzidic et al. 1975). Similarly, we proposed the following mechanism for ionization of PCNB with an APCI source in negative ion mode (Dalmia 2021; Dalmia et al. 2020).

$${\mathrm{O}}_2+{e}^{-}\to {\mathrm{O}}_2^{-}$$

$$M+{\mathrm{O}}_2^{-}\to {\left[M-\mathrm{Cl}+\mathrm{O}\right]}^{-}+\mathrm{ClO}$$

where *M* is PCNB.

**Fig. 4:**
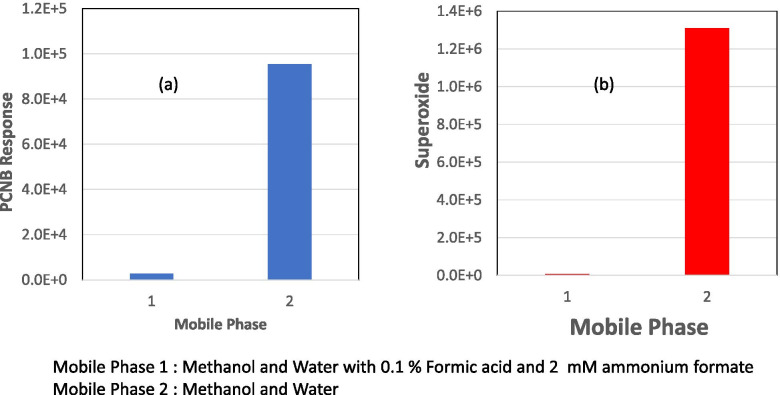
Effect of mobile phase composition on PCNB (**a**) and superoxide ion response (**b**). Both superoxide ion (O_2_^−^) and PCNB signal increased roughly by a factor of 300 and 30, respectively, when we changed the mobile phase from a mixture of methanol and water with 0.1% formic acid and 2 mM ammonium formate to a mixture of methanol and water. Mobile phase 1: methanol and water with 0.1% formic acid and 2 mM ammonium formate. Mobile phase 2: methanol and water

Herein, the formation of [M-Cl+O]^−^ can be attributed to the formation of superoxide ion (O_2_^−^) by electron capture followed by its chemical reaction with PCNB. This mechanism can be explained further by analyzing the mass spectra for PCNB with an APCI source. The mass spectra for PCNB showed a monoisotopic peak at a nominal mass of 274 dalton. The nominal monoisotopic mass of PCNB molecule is 293 dalton, and therefore, mass loss of 19 dalton from the molecule of PCNB can be explained by loss of chlorine (nominal monoisotopic mass of 35 dalton) and addition of oxygen ( nominal monoisotopic mass of 16 dalton) to PCNB molecule to form a negatively charged ion. Also, experimentally observed isotope pattern of PCNB ion matched very closely to theoretical isotope pattern of PCNB ion with four chlorine atoms, and this proved further that PCNB loses one chlorine atom in APCI ion source. We checked low mass spectra of the APCI ion source to confirm the formation of superoxide reagent ion species which could react with PCNB to ionize it. Figure 4 showed that both superoxide ion (O_2_^−^) and PCNB signal increased roughly by a factor of 300 and 30, respectively, when we changed the mobile phase from a mixture of methanol and water with 0.1% formic acid and 2 mM ammonium formate to mixture of methanol and water. This further proved that superoxide ion plays an important role in the ionization of PCNB in the APCI source.

### Proficiency testing

**Fig. 5:**
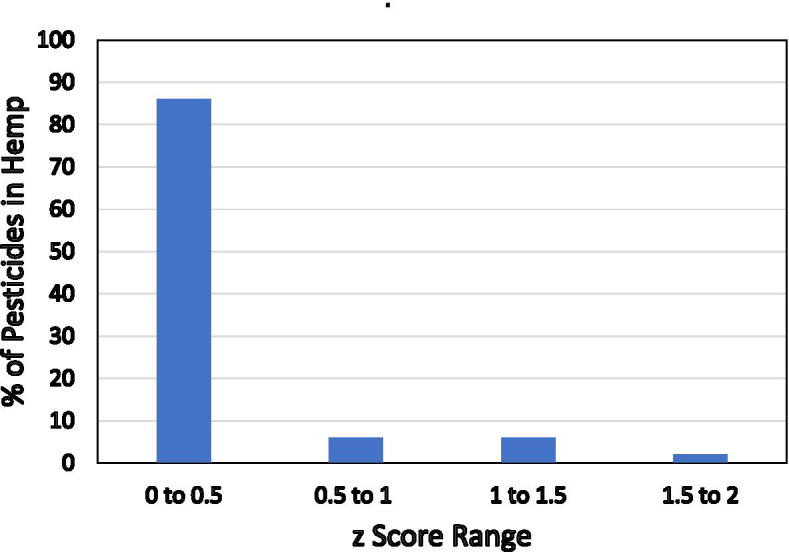
The distribution of *z* scores from the proficiency test report of 66 pesticides quantified in hemp sample using the LC-MS/MS method with ESI and APCI sources

The LC-MS/MS method generated satisfactory results since our proficiency test report showed *z* scores of less than 2 for all pesticides spiked in the hemp sample for proficiency testing. Figure 5 shows the distribution of *z* scores for pesticides quantified in the hemp matrix with our method. This figure shows that all *z* scores were less than the acceptable value of 2, and the majority (about 87%) of *z* scores were less than 0.5 which demonstrates excellent accuracy of our method for quantification of all of 66 pesticides in hemp. The proficiency test data did not report any false positive and false negative for 66 pesticides regulated by the California state in hemp.

### Stability studies

**Fig. 6:**
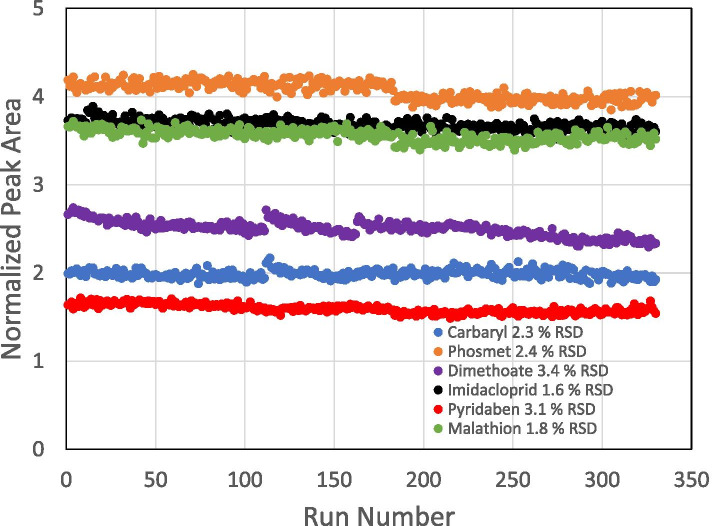
Long-term stability data over 5 days of 330 injections of six pesticides (carbaryl, phosmet, dimethoate, imidacloprid, pyridaben, and malathion) spiked in hemp using the LC-MS/MS method with an ESI source. The relative standard deviation (RSD) of signal for six analytes in hemp was less than 5% over a period of 5 days

Figure 6 shows the signal stability for 330 sample injections for six analytes (carbaryl, phosmet, dimethoate, imidacloprid, pyridaben, and malathion) over a period of 5 days. The percentage of RSDs of signal for all 66 analytes in hemp was less than 20%.

## Discussion

Currently, most published pesticide analysis methods deploy both LC-MS/MS and GC-MS/MS instruments and tedious sample preparation methods such as SPE and QuEChERS with dSPE to meet the low pesticide limits imposed by different USA states and countries such as Canada and others in cannabis matrices (Stenerson and Oden 2018; Kowlaski et al. 2017; Wang et al. 2016; Moulins et al. 2018). Herein, we presented an analytical LC-MS/MS method for a complete analysis of all 66 pesticides outlined in the California state regulations for cannabis-related products. This analytical LC-MS/MS method with dual ESI and APCI source and solvent extraction can be used for the analysis of pesticides regulated by different US states and countries such as Canada (Canada regulations for 96 pesticides in cannabis products 2019; Dalmia et al. 2019), Israel, and others in cannabis products. All the 66 pesticides were analyzed in hemp with dual-source mass spectrometer equipped with both APCI and ESI ionization probes. Pesticides such as methyl parathion, captan, cypermethrin, cyfluthrin, chlorfenapyr, chlordane, and pentochloronitrobenzene (quintozene), among others, which are conventionally analyzed by GC-MS/MS, were all detected with the LC-MS/MS system. A number of previous studies claimed that hydrophobic and non-polar pesticides (e.g., pentachloronitrobenzene, methyl parathion, chlordane, chlorfenapyr, captan, and others) are traditionally analyzed by GC-MS/MS since they do not ionize effectively by LC-MS/MS with an ESI source or APCI source. In this study, we demonstrated excellent detection limits in the range of 0.01–0.05 μg/g for chlorfenapyr, pentachloronitrobenzene, methyl parathion, and chlordane in hemp using LC-MS/MS with an APCI source. Previously, it was claimed that analysis of PCNB with an APCI source in LC-MS/MS is not selective and may require a quadratic calibration curve with a poor correlation coefficient (Curtis et al. 2019; Macherone 2019). However, this experimental work outlines a robust LC-MS/MS method with an APCI source which exhibits excellent sensitivity, selectivity, and linearity of PCNB analysis with APCI source. Similarly, in previous studies, the analysis of captan at low levels in different food matrices is not selective and reproducible using GC-MS/MS with an EI source due to the degradation of captan either in hot injector of GC or ion source (Dasgupta et al. 2010). Recently, atmospheric pressure gas chromatography (APGC) with tandem mass spectrometry (MS/MS) has been reported for the analysis of captan because their spectra exhibit far less in-source fragmentation than with an EI source (Cherta et al. 2013). Nevertheless, APGC does not address the degradation issues for captan in GC injector liner resulting in two peaks. In comparison with published GC-MS/MS methods for captan analysis, the LC-MS/MS method with an ESI source is quite sensitive and selective for captan analysis, since it achieves a low LOQ of 0.1 μg/g in hemp and uses MRM transition (316.9/263.9) based on intact molecular ion (ammonia adduct of captan) without incurring any degradation.

Solvent extraction is a quick, high-throughput, and easy way to achieve high extraction recovery in comparison with other time-consuming sample preparation techniques like solid-phase extraction (SPE) and QuEChERS with dSPE requiring multiple steps and large sample and solvent volumes and with low recoveries for some of the pesticides on the California list (Stenerson and Oden 2018; Kowlaski et al. 2017; Wang et al. 2016; Moulins et al. 2018; Alder et al. 2006; United States Department of Agriculture Food Safety and Inspection Service, Office of Public Health Science 2018; Anastassiades et al. 2003). The LC-MS/MS method described in this paper and proficiency test report demonstrated that it is possible to measure 66 pesticides on the California list in hemp using LC-MS/MS with ESI and APCI with good sensitivity, selectivity, and good recovery of pesticides using solvent extraction. In the future, we would need to collect data with more replicates to validate this method.

We demonstrated the ionization mechanism of PCNB using an APCI source, and we would need to carry out more experiments in the future to demonstrate the ionization mechanism of other pesticides such as chlordane, methyl parathion, and others with an APCI source. The stability studies demonstrated that the Qsight LC-MS/MS system would reduce maintenance downtime with this dirty and challenging hemp matrix. In order to demonstrate the stability of the instrument for a longer period, we would need to extend our stability studies to a time period longer than 5 days.

## Conclusions

This study demonstrates a unique, quantitative, rapid, and reliable LC-MS/MS method for the analysis of 66 pesticides residues in hemp samples. The proposed solvent extraction method is suitable for labs wanting to comply with the California regulations, as the recovery of all pesticides from a hemp matrix was in the acceptable range of 80–120% with RSD less than 20%. This method allowed the identification and quantification of all 66 pesticides at low levels (0.001 to 0.1 μg/g). We elucidated the ionization mechanism of PCNB with an APCI source and demonstrated that the analysis of PCNB is quite selective, sensitive, and linear with an APCI source. The ability to screen and quantitate all 66 pesticides, including the very hydrophobic and chlorinated compounds normally analyzed on a GC-MS/MS system, makes this method suitable for screening and quantitation of 66 pesticides in hemp with a single instrument. Excellent 5 days stability data showed that the Qsight LC-MS/MS system needs less maintenance with dirty hemp matrix. The proficiency test data showed excellent accuracy of our method in the quantification of all of pesticide residues in the hemp matrix with a single LC-MS/MS platform with dual ESI and APCI ion sources.

## Data Availability

The data is available from the corresponding author on reasonable request.

## Abbreviations

ESI: Electrospray ionization
APCI: Atmospheric pressure chemical ionization
LOQ: Limit of quantitation
LC: Liquid chromatography
MS: Mass spectrometry
GC: Gas chromatography
THC: Tetrahydrocannabinol
CBD: Cannabidiol
QuEChERS: Quick, easy, cheap, effective, rugged, and safe
dSPE: Dispersive solid-phase extraction
SPE: Solid-phase extraction
HSID: Hot source induced desolvation
SP: Superficially porous
RSD: Relative standard deviation
MRM: Multiple reaction monitoring
EI: Electron ionization
APGC: Atmospheric pressure gas chromatography
FDA: Food and Drug Administration
PCNB: Pentachloronitrobenzene

## References

[CR1] Alder L, Greulich K, Kempe G, Vieth B (2006). Mass Spec Rev.

[CR2] Anastassiades M, Lehotay SJ, Stajnbaher D, Schenk FJ (2003). J AOAC Int.

[CR3] Bioanalytical Method Validation Guidance for Industry. 2018, available from https://www.fda.gov/files/drugs/published/Bioanalytical-Method-Validation-Guidance-for-Industry.pdf.

[CR4] Canada regulations for 96 pesticides in cannabis products. 2019. https://www.canada.ca/en/public-health/services/publications/drugs-health-products/cannabis-testing-pesticide-list-limits.html.

[CR5] Chapter 5. Testing Laboratories Section 5313 Residual Pesticides, Bureau of Marijuana Control Proposed Text of Regulations, CA Code of Regulations, Title 16, 42; n.d. p. 23–6.

[CR6] Cherta L, Portoles T, Beltran J, Pitarch E, Mol JGJ, Fernandez H (2013). J Chromatogr A.

[CR7] Curtis M, Fausett E, Hale WA, Honnold R, Westland J, Stone PJ, Hollis JS, Macherone A (2019). Cannabis Sci Technol.

[CR8] Dalmia A (2021). US patent number 10914713.

[CR9] Dalmia A, Cudjoe E, Astill T, Jalali J, Weisenseel JP, Qin F, Murphy M, Ruthenberg T (2018). Cannabis Sci Technol.

[CR10] Dalmia A, Cudjoe E, Jalali J, Wu J, Hariri S, Guthrie M, Astill T, Schmidt C, Greenbaum M, Qin F (2019). A novel ESI and APCI LC/MS/MS analytical method for meeting the Canadian Cannabis Pesticide Residues Regulatory Requirements.

[CR11] Dalmia A, Johnson C, Hariri S, Jalali J, Cudjoe E, Kingstad J, Qin F (2020). Curr Trends Mass Spec.

[CR12] Dasgupta S, Bannerjee K, Patil SH, Ghaste M, Dhumal KN, Adsule PG (2010). J Chromatogr A.

[CR13] Dzidic I, Carroll DI, Stillwell RN, Hornig EC (1975). Anal Chem.

[CR14] Exhibit A, Table 3. Pesticide analytes and their action levels. Oregon Administrative Rules 333-007-0400; Oregon/gov/oha, effective 5/31/2017.

[CR15] https://www.brookings.edu/blog/fixgov/2018/12/14/the-farm-bill-hemp-and-cbd-explainer/ (n.d.).

[CR16] ISO-13528 (2005). Statistical methods for use in proficiency testing by inter-laboratory comparison.

[CR17] Klein TW, Newton CA (2007). Adv Exp Med Biol.

[CR18] Kowlaski J, Dahl JH, Rigdon A, Cochran J, Laine D, Fagras G (2017). LCGC.

[CR19] Macherone A. Tackle emerging cannabis regulations with confidence. Agilent application brief. 2019. available from https://www.agilent.com/cs/library/applications/application-cannabis-hemp-pesticide-6545-1290-infinity-5994-1127en-agilent.pdf.

[CR20] Makkar HPS, Strnad I, Mittendorfer J (2016). J Agri Food Chem.

[CR21] Mastovska K, Lehotay SJ, Chromatogr J. 2004; A,1040:259–272. 10.1016/j.chroma.2004.04.017 15230533

[CR22] McEwena CN, Larsen BS (2009). J Am Soc Mass Spectrometry.

[CR23] Moulins JR, Blais M, Montsion K, Tully J, Mohan W, Gagnon M, McRitchie T, Kwong K, Snider N, Blais DR (2018). J AOAC Int.

[CR24] Stenerson KK, Oden G (2018). Cann Sci Tech.

[CR25] Thompson M, Ellison SR, Wood R (2006). Pure Appl Chem.

[CR26] United States Department of Agriculture Food Safety and Inspection Service, Office of Public Health Science (2018). Screening for pesticides by LC/MS/MS and GC/MS/MS.

[CR27] Wang X, Mackowsky D, Searfoss J, Telepchak M (2016). LCGC.

